# Prison Administration and the Training of Officials.—V

**Published:** 1898-04-30

**Authors:** 


### Modern Sociology.

### PRISON ADMINISTRATION AND THE TRAINING OF OFFICIALS.—V.

In dealing with the subject of prison administration in these pages hitherto, we have been mainly advocating the systematic training of the officials who, in various different capacities, are placed in charge of convicted prisoners. We must now turn to another class of persons who have it in their power to influence the inmates of our gaols, although their number is at present unhappily somewhat limited. We allude to the visitors appointed by the Home Office to have free access to the prisoners, with the view of assisting them, both during their incarceration and after their release, to rise from their depths of degradation and depravity to a new and better life. It is not possible to overestimate the value of this assistance, judiciously given, to these outcasts during the period of their confinement within the prison walls. There are many excellent agencies for the benefit of discharged prisoners, such as the Prisoners' Aid Society, the Police-court Missions, and the band of good volunteers who wait for them at the prison gate on the day when their sentence expires to offer them breakfast and companionship for a time, in order to prevent them from resorting at once to the public-house, where some "pal" of their own is sure to be ready to treat them. All these do admirable work in their measure, for which we may well be thankful; but one and all have a common foe which acts against them with almost irresistible power, and that is the first breath of freedom, the first sense of liberty, which comes with a rush of delight on the released man or woman, making them feel that nothing on earth can deprive them of the ecstatic certainty that they are their own masters once more, and that no human being has a right to prevent them from doing just what they please with themselves either in the present or the future. If they prefer the public-house to the quiet breakfast, none can coerce their will, and it is almost invariably their impulse to shake off all benevolent advisers, and get out of their reach as quickly as possible. Within the prison walls matters are very different. The sight of a face that is not that of an officer—the sound of a voice that does not speak in command, is welcomed by them as an inestimable boon, relieving them temporarily from the monotony of prison life. The touch of human sympathy, the consciousness that they are in the presence of one who comes to them voluntarily as a friend, who is to derive no pecuniary advantage for services rendered, has a wonderful power in drawing forth their confidence and gaining a lasting influence over them. It is remarkable how strongly these poor outcasts feel the difference between paid and unpaid service, and how keenly they are alive to such distinctions. We could give instances in proof of this. They understand perfectly well that all the officers, including the chaplains, receive a proper equivalent for devoting all their time and labour to prison duties, and that the voluntary visitors are the only persons appointed to the prison who are not salaried or recompensed in any way. To their limited intelligence it makes all the difference in their appreciation of the work done for them. They know that it is from pure kindness and friendliness that the unpaid visitor spends hours with them in their dismal abode, listening to the tale of their woes, and therefore believe their efforts must spring from genuine and disinterested care for them, such as could not actuate those whose routine duty had a definite mercantile value. In this idea, no doubt, they are quite mistaken. As a rule, the officials, and most especially the chaplains, have a high-minded and hearty desire to benefit the prisoners, and as earnest a desire to assist them as any of the voluntary workers. But the prisoners will never be convinced of that. While their *idèe fixe* on the subject is to be deplored, it undoubtedly puts power in the hands of the unpaid visitors which is not possessed by anyone else approaching them. It will be seen from this fact how great the value of the voluntary work must be. To this unique friend the prisoners freely tell all their painful histories, and listen eagerly to any suggestions made as to their future career, when the term of their sentence is over. By reiterated conversations on these themes during their incarceration, and by the strong influence of the visitor, it is quite possible to obtain such a firm hold on the prisoners as will counteract the tremendous power exercised over them by the sense of freedom on release, so that when discharged they prove willing to submit themselves to schemes of reformation. We may mention in this connection that the Labour Homes of the Church Army are most useful in providing at once both work and shelter for released prisoners.

For female convicts there are homes of a different description, though not so many as we could wish, where they are cared for morally and physically. Our object at present, however, is to show how very important it is that volunteer visitors should be appointed to every prison in the country, instead of only to a few specially selected, as is the case at present. We see no reason, either, why the system should be limited to the appointment of female workers only. It would be of great use to the prison chaplains if they could have some earnest laymen working under their supervision, especially with a view to the disposal of the prisoners on their release. We think, also, that it would be a very valuable addition to the lady visitors' work if they were allowed to see the sick men in the infirmary at stated hours. Lastly, we must state that a certain amount of training is as necessary for these ladies as for all other officials. There have been cases where the mistakes and indiscretions of lady visitors have rendered it necessary that they should be withdrawn from their posts. There could, however, be no difficulty in their obtaining all the advice and instruction necessary from experienced workers who have been long engaged in the prisons as to the principles that should guide them. The most important of these are absolute loyalty to the authorities, strict obedience to the rules, and while giving hearty sympathy and efficient aid to the prisoners on their discharge, the avoidance of all sentimentality or glossing over the criminality of the deeds which have brought them within the prison walls.

